# Associations between environmental tobacco smoke exposure and oral health symptoms in adolescents

**DOI:** 10.1186/s12903-022-02440-7

**Published:** 2022-09-12

**Authors:** Na-Young Yoon, Il Yun, Yu Shin Park, Eun-Cheol Park

**Affiliations:** 1 Department of Public Health, Graduate School, Yonsei University, Seoul, Republic of Korea; 2 Institute of Health Services Research, Yonsei University, Seoul, Republic of Korea; 3 Department of Preventive Medicine, Yonsei University College of Medicine, 50 Yonsei-ro, Seodaemun-gu, Seoul, 03722 Republic of Korea

**Keywords:** Tobacco, Oral health, Adolescent health

## Abstract

**Background:**

Oral health condition in adolescence impacts the oral well-being throughout life. This study aimed to determine the association between environmental tobacco smoke (ETS) exposure and oral health in adolescents, using nationally representative data.

**Methods:**

Using data from the 2020 Korea Youth Risk Behavior Web-based Survey, we assessed self-reported data on ETS exposure and oral health symptoms in 37,591 non-smoking adolescents. The dependent variables were self-reported oral health symptoms of adolescents (tooth fracture, dental pain, and gum bleeding). ETS exposure was the primary independent variable. Chi-square tests and multivariable logistic regression analyses were performed to examine these relationships.

**Results:**

ETS exposure was positively associated with oral symptoms compared to no-ETS exposure in adolescents [boys, odds ratio (OR) 1.56, 95% confidence interval (CI) 1.46–1.66; girls, OR 1.50, 95% CI 1.41–1.60]; individuals with good oral health habits such as frequent tooth brushing [boys, three times or more a day, OR 1.38, 95% CI 1.24–1.53] and less soda consumption [girls, less than once a day, OR 1.73, 95% CI 1.29–2.33] had a weaker association. ETS exposure was positively associated with dental pain [boys, OR 1.55, 95% CI 1.45–1.66; girls, OR 1.50, 95% CI 1.41–1.60] and gum bleeding [boys, OR 1.43, 95% CI 1.29–1.58; girls, OR 1.32, 95% CI 1.21–1.44]; however, tooth fracture was significantly associated only in girls [OR 1.28, 95% CI 1.13–1.45].

**Conclusions:**

ETS in various environments is negatively associated with oral health in adolescents. This association could vary depending on health habits. Sophisticated policies to protect South Korean adolescents from ETS can be developed from these findings.

## Background

Oral health refers to the health of teeth, gums, and the entire orofacial system that allows people to smile, chew, and speak [1]. Oral health has a significant impact on the overall health and quality of life of an individual. Poor oral health is also associated with a higher risk of mortality, including major causes of death such as cardiovascular and respiratory diseases, and infections [2, 3]. Therefore, the oral health condition in adolescence could impact oral well-being throughout life.

Environmental tobacco smoke (ETS) exposure, also known as passive smoking, includes a mixture of exhaled main and side-stream smoke that pollutes the air surrounding the area of tobacco consumption [4]. Tobacco smoke contains a deadly mix of more than 7000 chemicals, hundreds of which are toxic, including formaldehyde, benzene, lead, and cadmium [5]. The World Health Organization reported that approximately 1.2 million annual deaths are the result of non-smokers being exposed to ETS [6].

Many studies have shown that ETS exposure is significantly associated with numerous diseases in adolescents. A study reported an association between ETS exposure measured by cotinine levels and metabolic syndrome in adolescents using the U.S. National Health and Nutrition Examination Survey [7]. According to a study conducted in Kuwait, asthma may be another negative health outcome in adolescents resulting from ETS exposure [8]. Another study showed an association between ETS exposure and depression among South Korean adolescents [9].

According to previous studies, tobacco smoke may affect the immune system and saliva flow, aggravating the oral health of an individual [10]. Most of the studies that examined the association between tobacco smoking and oral health focused on the direct effect of tobacco consumption on the smokers’ oral health [11, 12]. On the other hand, several recent studies have targeted the oral health of young children, which is mostly affected by parental ETS [13]. A previous study showed that parental smoking behavior is associated with caries in 5-year-old children [14]. Another study showed that children exposed to ETS had a high occurrence of enamel opacities, which increases the risk of dental caries [4].

However, previous studies that examined the association between ETS and health focused less on oral health in adolescents, which might be easily affected by the unhealthy behaviors among their peers, parents, community, as well as their own [15, 16]. Further, the ETS exposure location of adolescents might be more diverse than that of children as they spend a substantial amount of time outside home [17]. Furthermore, most studies to date that examine the association between ETS and oral health have been limited by small sample sizes and the difficulties of analyzing the effects of ETS exposure location of adolescents on various oral health symptoms.

Therefore, the present study aimed to determine the association between ETS exposure and the oral health symptoms in adolescents using a relatively large sample obtained from a national cross-sectional survey. After that, we further performed subgroup analysis according to the health behavior of adolescents as the association could vary depending on their oral health habits. The locations and frequencies of ETS exposure and the prevalence of three different oral health symptoms, i.e., tooth fracture, dental pain, and gum bleeding, were examined to specifically analyze the relationship.

## Methods

### Data

The data used in this study were obtained from the 2020 Korea Youth Risk Behavior Web-based Survey (KYRBWS) for adolescents aged 12–18 years. KYRBWS is a nationwide cross-sectional survey conducted by the Korea Disease Control and Prevention Agency (KDCA). The KYRBWS was a secondary dataset available in the public domain. And its data were de-identified to maintain respondents’ anonymity and confidentiality. This survey was approved by the Korean National Statistical Office (Approval No. 117058). The survey was conducted in accordance with the guidelines and regulations provided by the Institutional Review Board of the KDCA. Data is available for download from the KDCA website (https://www.kdca.go.kr/yhs/). Therefore, this present study did not require additional approval or prior consent from the Institutional Review Board.

The purpose of the KYRBWS survey is to examine the status of health behaviors of South Korean adolescents and identify health indicators for the formation and evaluation of health programs. The survey is conducted annually and anonymously, in approximately 400 high schools and middle schools. The data were collected from August to November 2020, and the total number of survey participants of 2020 KYRBWS was 54,948 (response rate of 94.9%) [18].

From the database, the following respondents were excluded: 6081 respondents (4234 boys and 1847 girls) who had smoked cigarettes such as conventional and electronic cigarettes, and heated tobacco products in their lifetime; 1447 adolescents (913 boys and 534 girls) whose teeth had been fractured due to exercise or accidents; and 9829 participants (6019 boys and 3810 girls) who did not agree to provide their household information. Finally, 37,591 samples (17,187 boys and 20,404 girls) were analyzed.

### Variables

The dependent variable was self-reported oral health symptoms. To assess oral health symptoms, the KYRBWS inquired about the experience of “tooth fracture,” “dental pain,” and “gum bleeding” through four different indications: “chipped or broken tooth”, “toothache when eating or drinking”, “throbbing and sore tooth”, and “sore and bleeding gums” for last 12 months. We classified those who had more than one oral health symptom as “symptom group”, and those who did not as “symptomless group”. We further classified the “symptom group” based on each oral symptom.

The primary independent variable in this study was ETS exposure. The participants were asked about the frequency of inhalation of tobacco smoked by others during the last 7 days inside their “home”, “school”, or “other places (shops, restaurants, shopping malls, concert halls, internet cafe, karaoke, etc.)”. The responses for each of the three locations were as follows: “no”, “1 day”, “2 days”, “3 days”, “4 days”, “5 days”, “6 days”, or “7 days”. Then, we classified those who had no ETS exposure at either location as “No ETS group”, and those who had exposure either inside home, school or other indoor places as “ETS group”. Subsequently, we divided the “ETS group” into subgroups based on location and frequency.

Other independent variables that may act as potential confounding variables include sex, age group (middle or high school), region, school grades, frequency of drinking soda and tooth brushing, quality of sleep, house affluence, type of household, and education of parents. Based on the exclusion criteria, only lifetime non-smoking adolescents were excluded. This was assessed by questions about lifetime smoking experience of conventional and electronic cigarettes, and heated tobacco products.

### Statistical analysis

Chi-square test and multivariable logistic regression analysis were used to analyze the data. The general characteristics of the sample were analyzed using frequencies and means. Multivariable logistic regression analyses were performed to examine the association between ETS exposure and oral health symptoms in adolescents after adjusting for control variables. The results are reported as odds ratios (OR) and confidence intervals (CI). A subgroup analysis was performed and stratified by sex and location of ETS exposure. All statistical analyses were performed using SAS software (version 9.4, SAS Institute, Cary, NC, USA).

## Results

We analyzed each variable according to sex. Table 1 presents the general characteristics of the participants. Among the 37,591 study participants, 17,187 (45.7%) were boys and 20,404 (54.3%) were girls. Based on the ETS exposure, the proportion of female students with oral health symptoms in the “ETS group” (57.3%) was higher than that of male students (48.5%). Table 2 shows the association between ETS exposure and oral health symptoms after adjusting for all confounding variables. Regardless of the sex, the ETS exposure group was significantly associated with oral health symptoms (boys, OR 1.56, 95% CI 1.46–1.66; girls, OR 1.50, 95% CI 1.41–1.60).

**Table 1:** General characteristics of the study population

Variables	Boys							Girls						
	Oral health symptoms							Oral health symptoms						
	Total		Normality		Abnormality		*P* value	Total		Normality		Abnormality		*P* value
	N	%	N	%	N	%		N	%	N	%	N	%	
Total (*N* = 37,591)	17,187	45.7	9983	58.1	7204	41.9		20,404	54.3	9627	47.2	10,777	52.8	
Environmental tobacco smoke (ETS) exposure							< 0.0001							< 0.0001
Yes	8246	48.0	4248	51.5	3998	48.5		12,598	61.7	5374	42.7	7224	57.3	
No	8941	52.0	5735	64.1	3206	35.9		7806	38.3	4253	54.5	3553	45.5	
Region							0.004							0.829
Metropolitan	7686	44.7	4537	59.0	3149	41.0		8546	41.9	4039	47.3	4507	52.7	
Urban	8177	47.6	4647	56.8	3530	43.2		10,188	49.9	4812	47.2	5376	52.8	
Rural	1324	7.7	799	60.3	525	39.7		1670	8.2	776	46.5	894	53.5	
Middle and high school							< 0.0001							< 0.0001
High school	6667	38.8	3705	55.6	2962	44.4		8769	43.0	3774	43.0	4995	57.0	
Middle school	10520	61.2	6278	59.7	4242	40.3		11,635	57.0	5853	50.3	5782	49.7	
Academic achievement							< .0.0001							<0.0001
High	6992	40.7	3953	56.5	3039	43.5		7707	37.8	3561	46.2	4146	53.8	
Middle	5149	30.0	3131	60.8	2018	39.2		6493	31.8	3222	49.6	3271	50.4	
Low	5046	29.4	2899	57.5	2147	42.5		6204	30.4	2844	45.8	3360	54.2	
Stress level							< 0.0001							<0.0001
High	4377	25.5	2156	49.3	2221	50.7		7915	38.8	3157	39.9	4758	60.1	
Middle	7778	45.3	4445	57.1	3333	42.9		9181	45.0	4511	49.1	4670	50.9	
Low	5032	29.3	3382	67.2	1650	32.8		3308	16.2	1959	59.2	1349	40.8	
Toothbrushing frequency in a day							< 0.0001							<0.0001
3 times or more a day	7208	41.9	4528	62.8	2680	37.2		10,955	53.7	5437	49.6	5518	50.4	
Twice a day	8173	47.6	4579	56.0	3594	44.0		8350	40.9	3784	45.3	4566	54.7	
None or once a day	1806	10.5	876	48.5	930	51.5		1099	5.4	406	36.9	693	63.1	
Soda intake frequency							0.015							<0.0001
More than once a day	1319	7.7	752	57.0	567	43.0		901	4.4	396	44.0	505	56.0	
1–6 times a week	12,629	73.5	7277	57.6	5352	42.4		13,802	67.6	6366	46.1	7436	53.9	
None	3239	18.8	1954	60.3	1285	39.7		5701	27.9	2865	50.3	2836	49.7	
Sleep time for fatigue recovery							< 0.0001							< 0.0001
Sufficient	6626	38.6	4364	65.9	2262	34.1		5521	27.1	3032	54.9	2489	45.1	
Normal	5852	34.0	3341	57.1	2511	42.9		6844	33.5	3362	49.1	3482	50.9	
Not sufficient	4709	27.4	2278	48.4	2431	51.6		8039	39.4	3233	40.2	4806	59.8	
Family affluence							0.000							0.032
High	4186	24.4	2355	56.3	1831	43.7		5687	27.9	2620	46.1	3067	53.9	
Middle	9402	54.7	5436	57.8	3966	42.2		11,084	54.3	5232	47.2	5852	52.8	
Low	3599	20.9	2192	60.9	1407	39.1		3633	17.8	1775	48.9	1858	51.1	
Having parents							0.531							0.921
Both parents	16,191	94.2	9395	58.0	6796	42.0		19,290	94.5	9103	47.2	10,187	52.8	
Single parent or none	996	5.8	588	59.0	408	41.0		1114	5.5	524	47.0	590	53.0	
Parents' education level							<0.0001							<0.0001
≤ Middle	132	0.8	83	62.9	49	37.1		164	0.8	71	43.3	93	56.7	
High school	3056	17.8	1747	57.2	1309	42.8		4297	21.1	1969	45.8	2328	54.2	
≥ College	10,716	62.3	6021	56.2	4695	43.8		13,119	64.3	6065	46.2	7054	53.8	
Unknown	3283	19.1	2132	64.9	1151	35.1		2824	13.8	1522	53.9	1302	46.1

**Table 2:** Association between ETS and oral health symptoms

Variables	Boys		Girls	
	Oral health symptoms		Oral health symptoms	
	OR	95% CI	OR	95% CI
Environmental tobacco smoke (ETS) exposure				
Yes	1.56	(1.46–1.66)	1.50	(1.41–1.60)
No	1.00		1.00	
Region				
Metropolitan	1.05	(0.93–1.20)	0.98	(0.84–1.13)
Urban	1.14	(1.00–1.30)	0.96	(0.83–1.11)
Rural	1.00		1.00	
Middle and high school				
High school	1.09	(1.01–1.17)	1.35	(1.26–1.44)
Middle school	1.00		1.00	
Academic achievement				
High	1.00		1.00	
Middle	0.85	(0.79–0.92)	0.84	(0.78–0.91)
Low	0.93	(0.85–1.02)	0.92	(0.84–0.99)
Stress level				
High	1.62	(1.47–1.79)	1.81	(1.64–2.00)
Middle	1.35	(1.25–1.47)	1.39	(1.27–1.52)
Low	1.00		1.00	
Toothbrushing frequency in a day				
3 times or more a day	1.00	(1.22–1.41)	1.00	
Twice a day	1.31		1.21	(1.12–1.30)
None or once a day	1.78	(1.58–2.02)	1.68	(1.45–1.95)
Soda intake frequency				
More than once a day	1.09	(0.94–1.26)	1.21	(1.03–1.42)
1–6 times a week	1.05	(0.96–1.15)	1.16	(1.09–1.25)
None	1.00		1.00	
Sleep time for fatigue recovery				
Sufficient	1.00		1.00	
Normal	1.26	(1.16–1.37)	1.11	(1.02–1.20)
Not sufficient	1.70	(1.55–1.86)	1.45	(1.34–1.57)
Family affluence				
High	1.16	(1.04–1.30)	1.08	(0.98–1.20)
Middle	1.13	(1.02–1.24)	1.05	(0.96–1.15)
Low	1.00		1.00	
Having parents				
Both parents	1.00		1.00	
Single parent or none	1.06	(0.90–1.24)	1.10	(0.95–1.27)
Parents' education level				
≤ Middle	1.00		1.00	
High school	1.36	(0.92–2.01)	0.80	(0.56–1.15)
≥ College	1.44	(0.98–2.13)	0.85	(0.59–1.21)
Unknown	1.07	(0.72–1.60)	0.66	(0.45–0.95)

Table 3 shows the results of the subgroup analyses between ETS exposure and oral health symptoms by covariates related to the following oral health management behaviors: “Tooth brushing frequency in a day” and “Soda intake frequency”. In male adolescents, tooth brushing for three or more times a day (OR 1.38, 95% CI 1.24–1.53) had a smaller association between ETS exposure and oral health symptoms than less frequent tooth brushing. In female adolescents, there was a stronger association in those who brushed their teeth less than once a day (OR 1.73, 95% CI 1.29–2.33) than in those who brushed their teeth more often. Furthermore, male adolescents who drank soda more than once a day (OR 2.11, 95% CI 1.66–2.69) had a greater association than less frequent drinkers. Female adolescents who did not drink soda (OR 1.33, 95% CI 1.18–1.50) had a lesser association with oral symptoms than more frequent drinkers.

**Table 3:** Results of subgroup analysis between ETS and oral health status by covariates

Variables	Boys			Girls		
	Oral health symptoms			Oral health symptoms		
	None	ETS exposure		None	ETS exposure	
	OR	OR	95% CI	OR	OR	95% CI
Toothbrushing frequency in a day						
3 times or more a day	1.00	1.38	(1.24–1.53)	1.00	1.51	(1.39–1.65)
Twice a day	1.00	1.73	(1.58–1.90)	1.00	1.47	(1.34–1.61)
None or once a day	1.00	1.59	(1.28–1.97)	1.00	1.73	(1.29–2.33)
Soda intake frequency						
More than once a day	1.00	2.11	(1.66–2.69)	1.00	1.52	(1.10–2.09)
1–6 times a week	1.00	1.52	(1.40–1.64)	1.00	1.59	(1.47–1.72)
None	1.00	1.55	(1.31–1.84)	1.00	1.33	(1.18–1.50)
Middle and high school						
High school	1.00	1.66	(1.51–1.83)	1.00	1.54	(1.41–1.68)
Middle school	1.00	1.49	(1.36–1.63)	1.00	1.47	(1.35–1.61)
Family affluence						
High	1.00	1.57	(1.36–1.80)	1.00	1.40	(1.24–1.59)
Middle	1.00	1.55	(1.42–1.68)	1.00	1.49	(1.37–1.63)
Low	1.00	1.58	(1.35–1.84)	1.00	1.75	(1.49–2.04)
Parents' education level						
≤ Middle	1.00	1.52	(0.68–3.42)	1.00	1.72	(0.74–3.97)
High school	1.00	1.79	(1.52–2.11)	1.00	1.66	(1.44–1.91)
≥ College	1.00	1.54	(1.42–1.67)	1.00	1.45	(1.34–1.57)
Unknown	1.00	1.43	(1.21–1.70)	1.00	1.59	(1.34–1.88)

Table 3 also reports the result of subgroup analyses between ETS exposure and oral health symptoms by demographic and socioeconomic factors of respondents. In female adolescents, when respondents’ family affluence was in low group, the association between ETS exposure and their oral health symptoms were stronger than in middle, and high group [low, OR 1.75, 95% CI 1.49–2.04; middle, OR 1.49, 95% CI 1.37–1.63; high, OR 1.40, 95% CI 1.24–1.59]. Likewise, in female adolescents, when their parents’ education level was lower, the association between ETS exposure and oral health symptoms was stronger [≤ middle, OR 1.72, 95% CI 0.74–3.97; high school, OR 1.66, 95% CI 1.44–1.91; ≥ college, OR 1.45, 95% CI 1.34–1.57]. On the other hand, those relationship according to family affluence and parents’ education level was not conspicuously shown in male adolescents.

In Table 3, we further analyzed the association according to age (middle school/high school) of respondents. The result showed that the association between ETS and their oral health symptoms were stronger when adolescents were older [boys, middle school, OR 1.49, 95% CI 1.36–1.63; high school, OR 1.66, 95% CI 1.51–1.83; girls, middle school, OR 1.47, 95% CI 1.35–1.61; high school, OR 1.54, 95% CI 1.41–1.68].

Table 4 shows the results of the subgroup analysis stratified by primary independent variables. Table 4 reports the OR for oral health symptoms according to ETS location and frequency. The groups that reported no ETS exposure at home, school, and other places were set as the reference groups. Notably, in male adolescents, ETS exposure at home of 7 days was more strongly associated with oral symptoms than exposure for 1–3 days (1–3 days, OR 1.22, 95% CI 1.11–1.33; 7 days, OR 1.34, 95% CI 1.15–1.56). In female adolescents, ETS exposure at home of more than 4–6 days was more strongly associated with having oral symptoms than exposure for 1–3 days (1–3 days, OR 1.13, 95% CI 1.03–1.23; 4–6 days, OR 1.22, 95% CI 1.06–1.42; 7 days, OR 1.18, 95% CI 1.03–1.34). Furthermore, only girls had a significantly positive association with oral symptoms after 1–3 days of ETS exposure at school (OR 1.30, 95% CI 1.12–1.51). In male adolescents, ETS exposure of more than 4–6 days at other indoor places during the last 7 days, was more strongly associated with oral symptoms, as compared to ETS exposure of 1–3 days of ETS (1–3 days, OR 1.42, 95% CI 1.32–1.54; 4–6 days, OR 1.84, 95% CI 1.56–2.17; 7 days, OR 1.72, 95% CI 1.32–2.23). Female adolescents were more strongly associated with oral symptoms in case of 7 days of ETS exposure than its lesser frequency at other indoor places (1–3 days, OR 1.39, 95% CI 1.30–1.49; 4–6 days, OR 1.70, 95% CI 1.50–1.93; 7 days, OR 1.88, 95% CI 1.60–2.20).

**Table 4:** Results of subgroup analysis stratified by interesting variable

Variables	Boys		Girls	
	Oral health symptoms		Oral health symptoms	
	OR	95% CI	OR	95% CI
Home ETS				
None	1.00		1.00	
1–3 days	1.22	(1.11–1.33)	1.13	(1.03–1.23)
4–6 days	1.17	(0.99–1.39)	1.22	(1.06–1.42)
7 days	1.34	(1.15–1.56)	1.18	(1.03–1.34)
School ETS				
None	1.00		1.00	
1–3 days	1.14	(0.98–1.34)	1.30	(1.12–1.51)
4–6 days	0.95	(0.64–1.42)	0.94	(0.64–1.38)
7 days	1.03	(0.66–1.61)	1.48	(0.88–2.48)
Else ETS				
None	1.00		1.00	
1–3 days	1.42	(1.32–1.54)	1.39	(1.30–1.49)
4–6 days	1.84	(1.56–2.17)	1.70	(1.50–1.93)
7 days	1.72	(1.32–2.23)	1.88	(1.60–2.20)

**Fig. 1:**
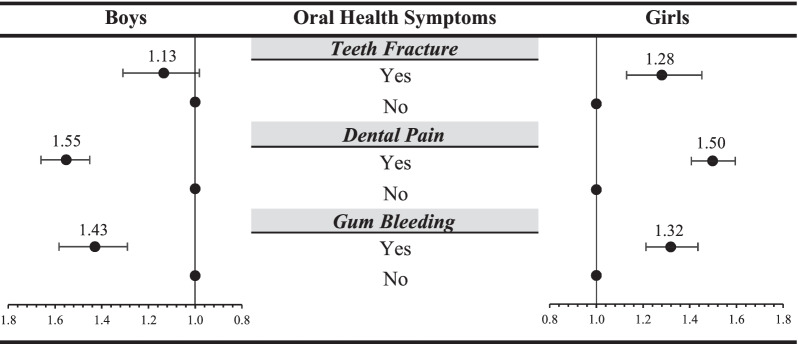
Results of subgroup analysis stratified by dependent variable

Figure 1 presents the OR for the three oral health symptoms. In male adolescents, there was a positive association between ETS experience and dental pain (OR 1.55, 95% CI 1.45–1.66) and gum bleeding (OR 1.43, 95% CI 1.29–1.58). In female adolescents, there was a positive association between ETS experience and all three oral symptoms: tooth fracture (OR 1.28, 95% CI 1.13–1.45), dental pain (OR 1.50, 95% CI 1.41–1.60), and gum bleeding (OR 1.32, 95% CI 1.21–1.44).

## Discussion

Most studies on ETS exposure of non-smoking adolescents have focused on the prevalence of respiratory or mental diseases. However, we aimed to determine the association between ETS and oral health status in adolescents. The results of this study found that non-smoking adolescents who experienced ETS exposure more than once had various oral symptoms regardless of the sex.

The mechanism behind the association between ETS and tooth fracture can be explained by the results of previous studies. The messenger RNA expression of dentin matrix acidic phosphoprotein-1, bone sialoprotein, and alkaline phosphatase activity were significantly decreased in nicotine-treated human dental pulp cells, and mineralized nodule formation was also inhibited by nicotine in human dental pulp cell [19, 20]. Even non-smokers show similar level of nicotine in their bodies when exposed to ETS for a long time [21], therefore, the functions of dentin matrix synthesis and mineralization may be decreased in the dental pulp cells of adolescents who exposed by ETS, which could lead to their tooth fracture.

Also, it is reported that ETS exposure may lead to a decrease in saliva flow rate and salivary α-amylase activity along with an increase in peroxidase activity, indicating the incidence of oxidative stress [22]. One study explained that ETS exposure could lead to elevation of interleukin-1beta, albumin, and aspartate aminotransferase levels in saliva [23]. Considering these mechanisms, abnormal oral health symptoms of dental pain, and gum bleeding in non-smoking adolescents could result from ETS exposure.

The present study also reports that the association between ETS exposure and oral health is statistically significant even when adolescents have different oral health management behaviors. However, the adverse effects of ETS exposure on oral health symptoms of adolescents can be reduced by good health habits, such as brushing teeth more often and consuming soda less frequently.

Previous studies have shown that adolescents who brush their teeth twice or more a day have significantly lower incidence of caries and counts of decayed, missing, or filled teeth [24]. These preventive oral health behaviors may lower the effects of ETS exposure. Lifestyle factors such as drinking soda also lead to negative oral health symptoms. Increased soda consumption is significantly associated with the prevalence of dental erosion, according to a previous study [25, 26].

Additionally, those results about the association between ETS exposure and oral health symptoms by socioeconomic status of female adolescents also can be supported by studies which explained that socioeconomic status could affect women’s inflammation, and immune function [27, 28]. Also, the result of stronger association between ETS exposure and oral health symptoms in older adolescents might be result of the cumulative effect, in that, bad oral health is progressive in nature [29, 30], and negative effects of ETS also becomes cumulative [31, 32]. As adolescents age, they might become more vulnerable to ETS.

Additional subgroup analyses of the locations and frequencies of ETS exposure confirmed that there was a statistically significant occurrence of oral health symptoms in both sex groups when they experienced ETS exposure at home. Furthermore, frequent ETS exposure at other indoor locations was associated with oral health symptoms. However, a significant association between frequent ETS exposure at school and oral health symptoms when compared with non-school ETS exposure, was observed only in female adolescents.

A previous study with a purpose similar to that of our study showed that children of parents who smoked a higher number of cigarettes reported higher cotinine concentrations than children of non-smoking parents [13]. Furthermore, another study showed that adults who experienced ETS exposure for more than two hours per day had a higher risk of cardiovascular disease than adults who experienced it for less than two hours per day [33]. As shown in these studies, the frequency of ETS could become an important factor that determines the wellness of individuals who are exposed to ETS.

Finally, the present study examined the association between ETS exposure and the prevalence of these three oral symptoms. There was a significant association between ETS and dental pain and gum bleeding in the male adolescent group and tooth fracture, dental pain, and gum bleeding in the female adolescent group. These results based on sex differences are similar to those of several previous studies, which show a stronger association of ETS with numerous diseases in the female group than in the male group; however, this should be interpreted cautiously and investigated further [34, 35].

This study has several limitations that should be considered. First, cross-sectional data were used. Therefore, the association between variables could be confirmed; causality could not be determined. Second, the results were derived from self-reported data. We specifically assessed the oral health symptoms of an individual, ETS frequencies, socioeconomic status, and health behavior covariates based on self-reported data. This finding may have been subject to recall bias [36]. Hence, the data may not have been accurately measured and may not be reliable. To provide more reliable results, future research should be conducted using the results of clinical examinations to assess the oral health status, and assessment of biological biomarkers such as salivary/blood cotinine levels to substantiate the results from self-reported data. Third, there might be factors such as individual lifestyle and personal traits, which co-vary with the oral symptoms of an adolescent and are not considered in this research model.

Despite these limitations, our study has several strengths. First, we used nationally representative data that were suitable for generalizing the results of the study to the overall South Korean adolescent population in middle and high schools. Furthermore, KYRBWS is an anonymous web-based survey that is likely to obtain relatively honest responses [37]. Third, in South Korea, few studies have been performed on the associations between ETS and oral health of adolescents, which analyzes these relationships in multi-dimensional aspects.

## Conclusion

Our study is meaningful because it reflects the current ETS patterns of non-smoking South Korean adolescents and their association with oral symptoms. The findings of our study emphasize the importance of protecting adolescents from ETS in various environments. Multi-dimensional aspects of ETS exposure of adolescents and health habits should be considered when developing sophisticated health policies. The results of this study can be used as a baseline for developing effective policies to protect South Korean adolescents from ETS exposure.

## Data Availability

Publicly available datasets were analyzed in this study. These data can be found here: [https://www.kdca.go.kr/yhs] (accessed on 13 July 2022).

## Abbreviations

ETS: Environmental tobacco smoke
KYRBWS: Korea Youth Risk Behavior Web-based Survey
KDCA: Korea Disease Control and Prevention Agency
OR: Odds ratio
CI: Confidence interval
